# Fulvic acid ameliorates drought stress-induced damage in tea plants by regulating the ascorbate metabolism and flavonoids biosynthesis

**DOI:** 10.1186/s12864-020-06815-4

**Published:** 2020-06-18

**Authors:** Jianhao Sun, Chen Qiu, Yiqian Ding, Yu Wang, Litao Sun, Kai Fan, Zhongshuai Gai, Guoqiang Dong, Jiguo Wang, Xinghui Li, Lubin Song, Zhaotang Ding

**Affiliations:** 1 Tea Research Institute, Qingdao Agricultural University, Qingdao, 266109 Shandong China; 2 Murdoch University, Perth, 6150 Australia; 3 The Quality and Safety Center of Agricultural Products, Qingdao, 266000 Shandong China; 4 The Service Center of Agricultural Technology, Rizhao, 276800 Shandong China; 5 Tea Research Institute, Nanjing Agricultural University, Nanjing, 210095 Jiangsu China; 6 Institue of Pomology, Shandong Academy of Agricultural Sciences, Taian, 271000 Shandong China

**Keywords:** *Camellia sinensis*, Fulvic acid, Drought stress, Ascorbate and glutathione metabolism, Flavonoids biosynthesis

## Abstract

**Background:**

Fulvic acid (FA) is a kind of plant growth regulator, which can promote plant growth, play an important role in fighting against drought, improve plant stress resistance, increase production and improve quality. However, the function of FA in tea plants during drought stress remain largely unknown.

**Results:**

Here, we examined the effects of 0.1 g/L FA on genes and metabolites in tea plants at different periods of drought stress using transcriptomics and metabolomics profiles. Totally, 30,702 genes and 892 metabolites were identified. Compared with controlled groups, 604 and 3331 differentially expressed metabolite genes (DEGs) were found in FA-treated tea plants at 4 days and 8 days under drought stress, respectively; 54 and 125 differentially expressed metabolites (DEMs) were also found at two time points, respectively. Bioinformatics analysis showed that DEGs and DEMs participated in diverse biological processes such as ascorbate metabolism (*GME*, *AO, ALDH* and L-ascorbate), glutathione metabolism (*GST*, *G6PDH*, glutathione reduced form and CYS-GYL), and flavonoids biosynthesis (*C4H*, *CHS*, *F3’5’H*, *F3H*, kaempferol, quercetin and myricetin). Moreover, the results of co-expression analysis showed that the interactions of identified DEGs and DEMs diversely involved in ascorbate metabolism, glutathione metabolism, and flavonoids biosynthesis, indicating that FA may be involved in the regulation of these processes during drought stress.

**Conclusion:**

The results indicated that FA enhanced the drought tolerance of tea plants by (i) enhancement of the ascorbate metabolism, (ii) improvement of the glutathione metabolism, as well as (iii) promotion of the flavonoids biosynthesis that significantly improved the antioxidant defense of tea plants during drought stress. This study not only confirmed the main strategies of FA to protect tea plants from drought stress, but also deepened the understanding of the complex molecular mechanism of FA to deal with tea plants to better avoid drought damage.

## Background

Tea plant (*Camellia sinensis* (L.) O. Kuntze) is an evergreen crop that likes a humid climate. In field conditions, tea plants are often exposed to drought stress, which has profound effects on tea yield and quality [1]. With the shortage of water resources, the drought has become a main disturbance for tea production in fields, resulting in reduction of yield by around 14–40% in different cultivation areas every year [2, 3]. In addition, catechins, caffeine, theanine and some free amino acids in tea would be significantly reduced under drought stress, resulting in a serious decline in tea quality [4]. Therefore, exploring the response mechanisms of tea plants to drought stress is essential for developing and breeding new drought-resistant varieties.

Recently, the researches on drought-resistance mechanisms of tea plants have made some progress. In terms of plant morphology, physiology and biochemistry, drought stress could induce stomatal closure, promote the accumulation of proline, glycine betaine, glucose and fructose, and improve the activities of superoxide dismutase and peroxidase [5–8]. In terms of molecular response mechanisms, many drought-responsive genes have been found in tea plants, including heat-shock proteins, glutathione S-transferase genes, sucrose synthases, cytoskeleton proteins and transcription factors [9–12]. However, due to the late start of the study on the mechanism of tea drought resistance, there were few studies on the effects of humic acid application on tea drought resistance under drought stress.

Fulvic acid (FA), an active humic substance, has a relatively low molecular weight and contains a high amount of oxygen-rich and carbon-poor functional groups [13, 14]. Many studies have shown that FA has many plant physiological activities. FA could significantly alleviate the toxic symptoms of Cd on lettuce seedlings [15].. And FA could protect soybean and barley against salt stress [16, 17]. Moreover, FA played important roles in the improvement of drought resistance in plants. Anjum [18] reported that exogenous FA conferred drought tolerance to maize under drought stress by promoting proline contents. And the study of Ramin Lotfi [19] reported that exogenous FA substantially ameliorated the adversities of drought by enhanced levels of superoxide dismutase (*SOD*), peroxidase (*POD*) and catalase (*CAT*) in rapeseed plants. Regrettably, although these data were valuable for exploring the effects of FA under drought stress, no information had yet been provided on regulatory mechanisms in these studies. It is noteworthy whether the FA could modulate the drought tolerance of tea plants during drought stress.

In the present study, we hypothesis that FA could play positive roles in drought tolerance of tea plants by better enhancing the expression of specific genes and metabolites during drought stress. For this, we examined the effects of 0.1 g/L FA on genes and metabolites in tea plants at different periods of drought stress using transcriptomics and metabolomics profiles. These data were valuable for understanding of when and how much antioxidant metabolism should be altered to improve the performance of FA-treated tea plants under drought stress, and would serve as new guiding strategies for improving drought management systems of tea garden.

## Results

### Phenotypic and physiological responses of FA-treated tea plants under drought stress

**Fig. 1:**
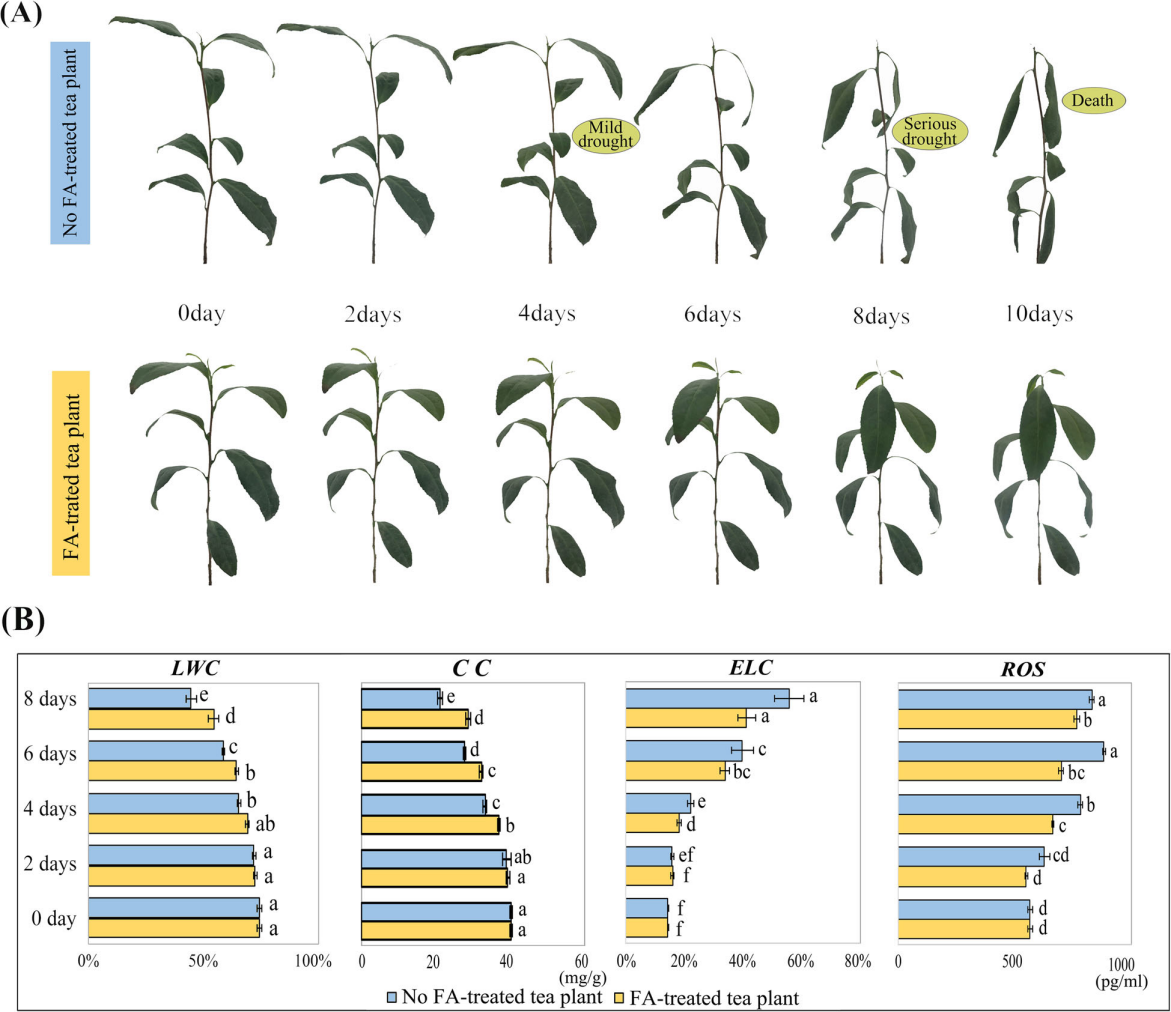
Phenotypic and physiological traits of FA-treated tea plants under drought stress. **a**. Phenotypes of FA-treated tea plants and the controlled groups. **b**. The value of *LWC* (leaf water content), *CC* (chlorophyll content), *ELC* (relative electrolyte conductivity) and *ROS* (reactive oxygen species)

In order to verify the effect of FA on tea plants during drought stress, we analyzed the phenotypic and physiological traits of tea shoots under drought stress. The tea plants exhibited the first visible symptom of drought damage, the wilting of shoots, after 4 days in controlled groups (without FA-treated tea plants), while FA-treated tea plants remarkably delayed the wilting of tea shoots. As well as after 8 days of drought, although FA-treated tea plants showed similar trend, the degree of wilting was lower than that of the control (Fig. 1a). Furthermore, the *LWC* (leaf water content), *CC* (chlorophyll content), *ELC* (relative electrolyte conductivity) and *ROS* (reactive oxygen species) in FA-treated tea plants changed significantly at 4 days of drought stress compared with controlled groups (Fig. 1b). Among them, the *LWC* and *CC* in FA-treated tea plants showed downward trends during drought stress, but the extents of the decrease were lower than that of the controlled groups. Furthermore, the *ELC* and *ROS* in FA-treated tea plants showed upward trends during drought stress, but the extents of the increase were lower than that of the controlled groups. In addition, after 8 days of drought stress, the differences of *LWC*, *CC*, *ELC* and *ROS* were largest between FA-treated groups and controlled groups. The results showed that the tea shoots at 4 and 8 days directly reflected the turning point of degree of drought stress. At this time, according to the data of the *WHC* (water holding capacity of soil), the tea plants were in a state of mild drought (65–75% *WHC*) and severe drought (15–25% *WHC*), respectively (Additional file 1). So, we selected the tea shoots of FA-treated groups and controlled groups at 4 days (mild drought) and 8 days (serious drought) as materials to perform transcriptomics and metabolomics analysis.

### The transcriptome analysis of FA-treated tea plants under drought stress

To explore the molecular events of 0.1 g/L FA-treated tea plants under drought stress, the transcriptome analysis was performed on tea shoots treated with FA (FAD) and without FA (WD) under drought stress. The samples of 4FAD, 4WD, 8FAD and 8WD were collected and sequenced (Additional file 2). The “4” and “8” represented the days of drought stress. After removing the low-quality reads, a total of 537,625,170 clean reads were obtained. The percentages of Q30 and GC were 93.785–95.22% and 44.38–44.83%, respectively, indicating that the quality of transcriptome sequencing data is high. A total of 30,702 genes were functionally annotated in the databases (Additional file 3). Moreover, 604 (227 up- and 377 down-regulated) and 3331 (2149 up- and 1182 down-regulated) DEGs (|fold change| > 2 and corrected *p*-value < 0.05) were identified in the pairwise comparison of 4FADvs4WD and 8FADvs8WD, respectively (Additional file 4). The results indicated that FA could induce the changes of transcripts in tea shoots under drought stress.

### The functional analysis of DEGs of FA-treated tea plants under drought stress

**Fig. 2:**
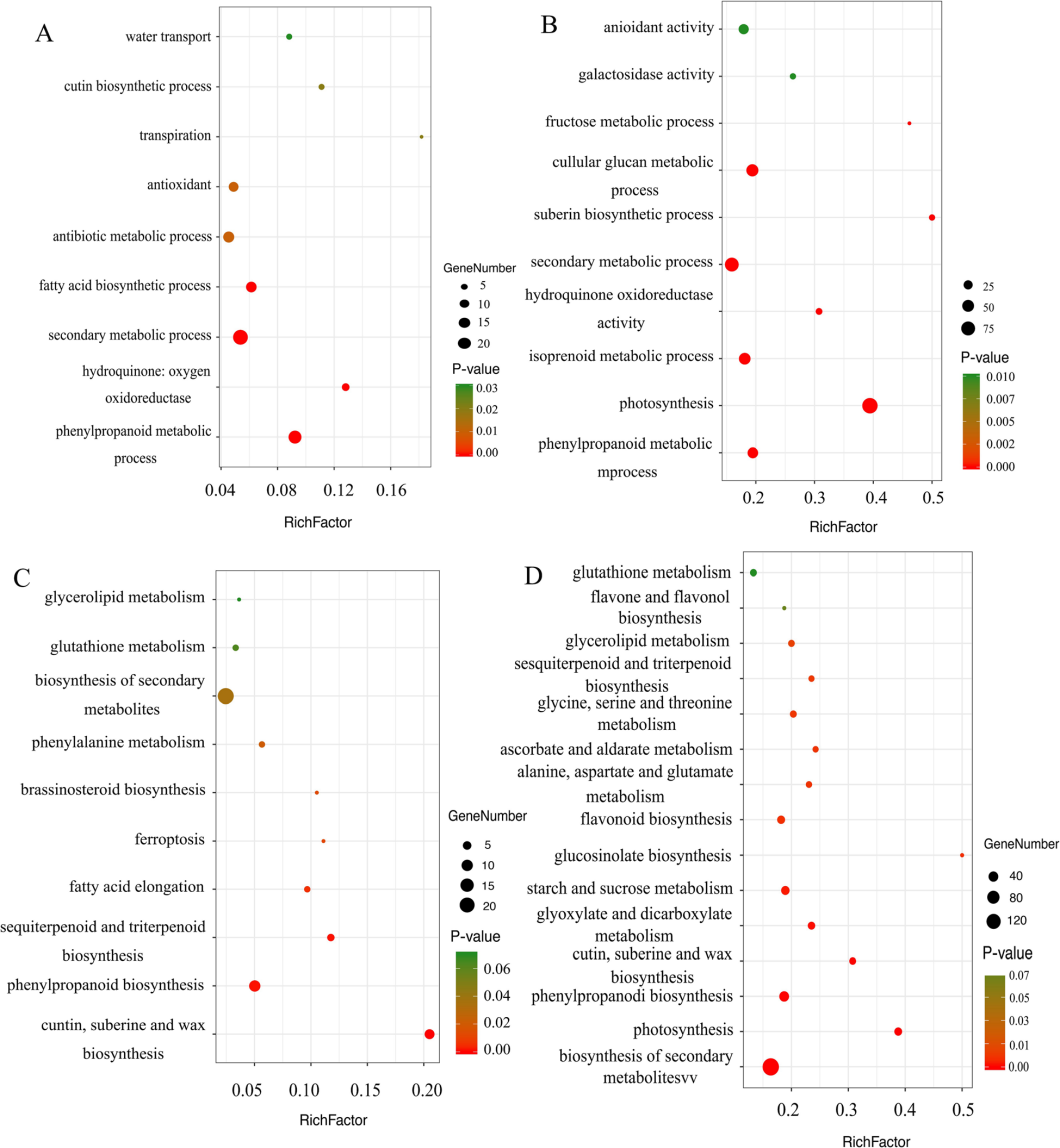
DEGs enriched on different GO terms and KEGG pathways. **a**. GO terms of DEGs in 4FADvs4WD. **b**. GO terms of DEGs in 8FADvs8WD. **c**. KEGG pathway analysis of DEGs in 4FADvs4WD. **d**. KEGG pathway analysis of DEGs in 8FADvs8WD

To further assess biological functions of DEGs in FA-treated tea plants under drought stress, the Gene Ontology (GO) and Kyoto Encyclopedia of Genes and Genomes (KEGG) enrichment analysis of DEGs were performed (Additional file 5). The results showed that the significantly enriched GO terms of DEGs in 4FADvs4WD were ‘secondary metabolic process’, ‘antioxidant activity’, ‘fatty acid biosynthetic process’ and ‘water transport’ (Fig. 2a). However, except for ‘secondary metabolic process’ and ‘antioxidant activity’, the GO terms of DEGs in 8FADvs8WD were mainly enriched in ‘isoprenoid metabolic process’, ‘fructose metabolic process’ and ‘galactosidase activity’ (Fig. 2b).

The KEGG pathways of DEGs in 4FADvs4WD and 8FADvs8WD were similar and both included ‘phenylpropanoid biosynthesis’, ‘glutathione metabolism’ and ‘cutin, suberine and wax biosynthesis’ (Fig. 2c and d). In addition, the pathways of DEGs in 4FADvs4WD were also highly enriched in ‘phenylalanine metabolism’ and ‘fatty acid elongation’. While, the high represented pathways of DEGs in 8FADvs8WD were ‘flavonoid biosynthesis’, ‘ascorbate and aldarate metabolism’, ‘starch and sucrose metabolism’ and ‘photosynthesis’. The results indicated that FA could regulate the complex biological pathways of tea plants under drought stress.

### The metabolome analysis of FA-treated tea plants under drought stress

**Fig. 3:**
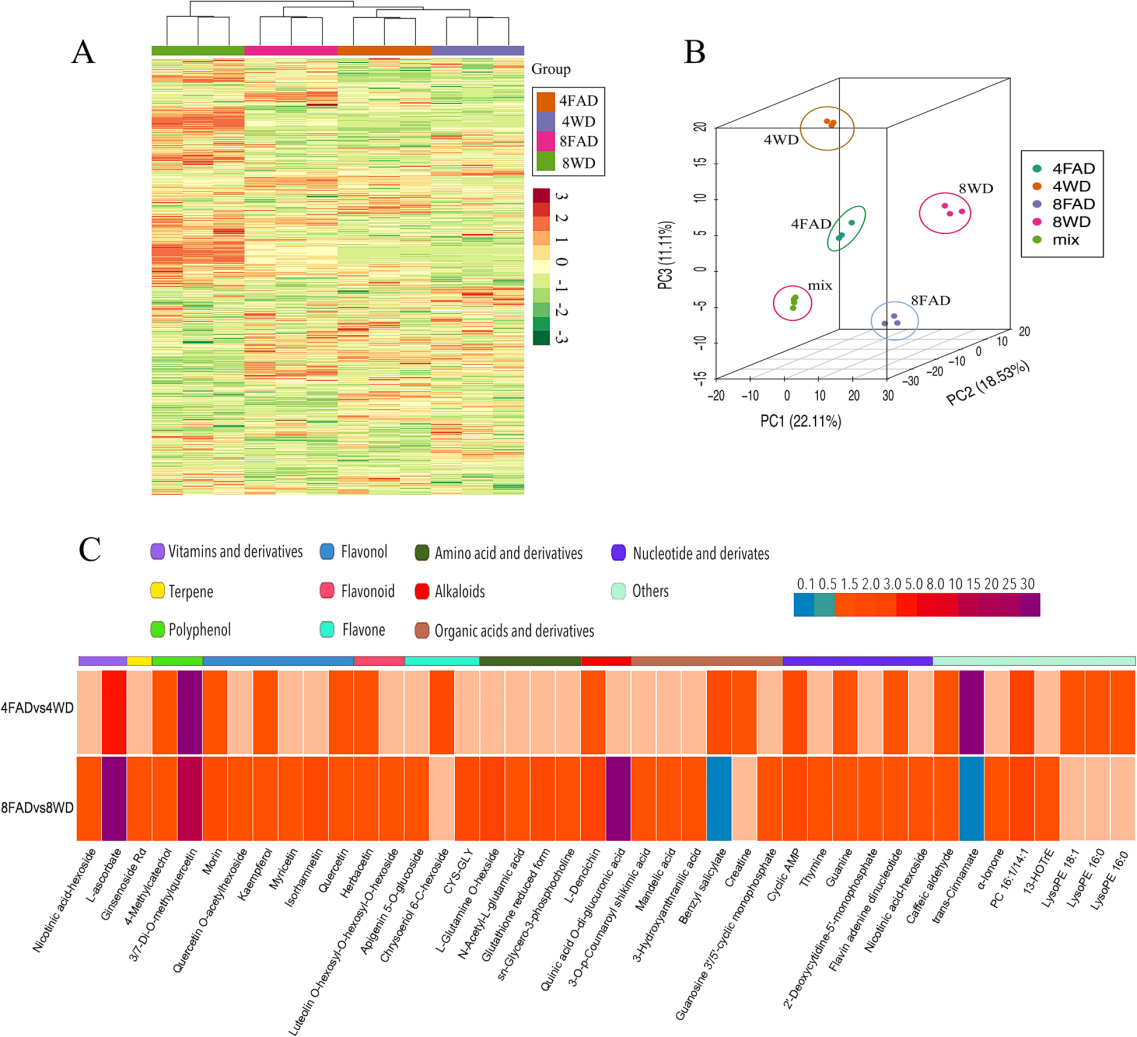
The metabolites analysis in tea plants under drought stress. **a**: Heat map visualization of metabolites. The content of each metabolite was normalized to complete linkage hierarchical clustering. Each example is visualized in a single column and each metabolite is represented by a single row. Red indicates high abundance, whereas low relative metabolites are shown in green (color key scale right of the heat map). **b**: PCA analysis of metabolites. **c**: The accumulated metabolites in 4FADvs4WD and 8FADvs8WD, respectively

To further analyze the metabolites of FA-treated tea plants under drought stress, the analysis of widely targeted metabolome was performed using LC-ESI-MS/MS system. A total of 891 metabolites were obtained in all samples (Fig. 3a, Additional file 6), and clearly separated into four groups according to the PCA analysis (Fig. 3b). Based on a fold-change threshold > 1.5, the numbers of up-regulated and down-regulated metabolites in 4FADvs4WD and 8FADvs8WD were 21 and 32, 34 and 90, respectively (Additional file 7). These differentially expressed metabolites (DEMs) were summarized into 143 species, which were mainly classified into flavonoid (2), vitamins and derivatives (3), flavonol (6), flavone (7), phenylpropanoids (15), organic acids and derivatives (24) and others (Additional file 8). Interestingly, the DEMs associated with osmotic adjustment, such as proline, glucose and fructose, were not found to accumulate significantly in 4FADvs4WD and 8FADvs8WD. However, whether in 4FADvs4WD or in 8FADvs8WD, the DEMs related to ascorbate metabolism and flavonoids biosynthesis were all highly accumulated. In addition, the more DEMs related to flavonoids biosynthesis were found in 8FADvs8WD and the DEMs related to glutathione metabolism were also accumulated (Fig. 3c). The results showed that FA could induce the changes of antioxidants in tea plants under drought stress.

### The co-expression network analysis of DEGs and DEMs in FA-treated tea plants under drought stress

**Fig. 4:**
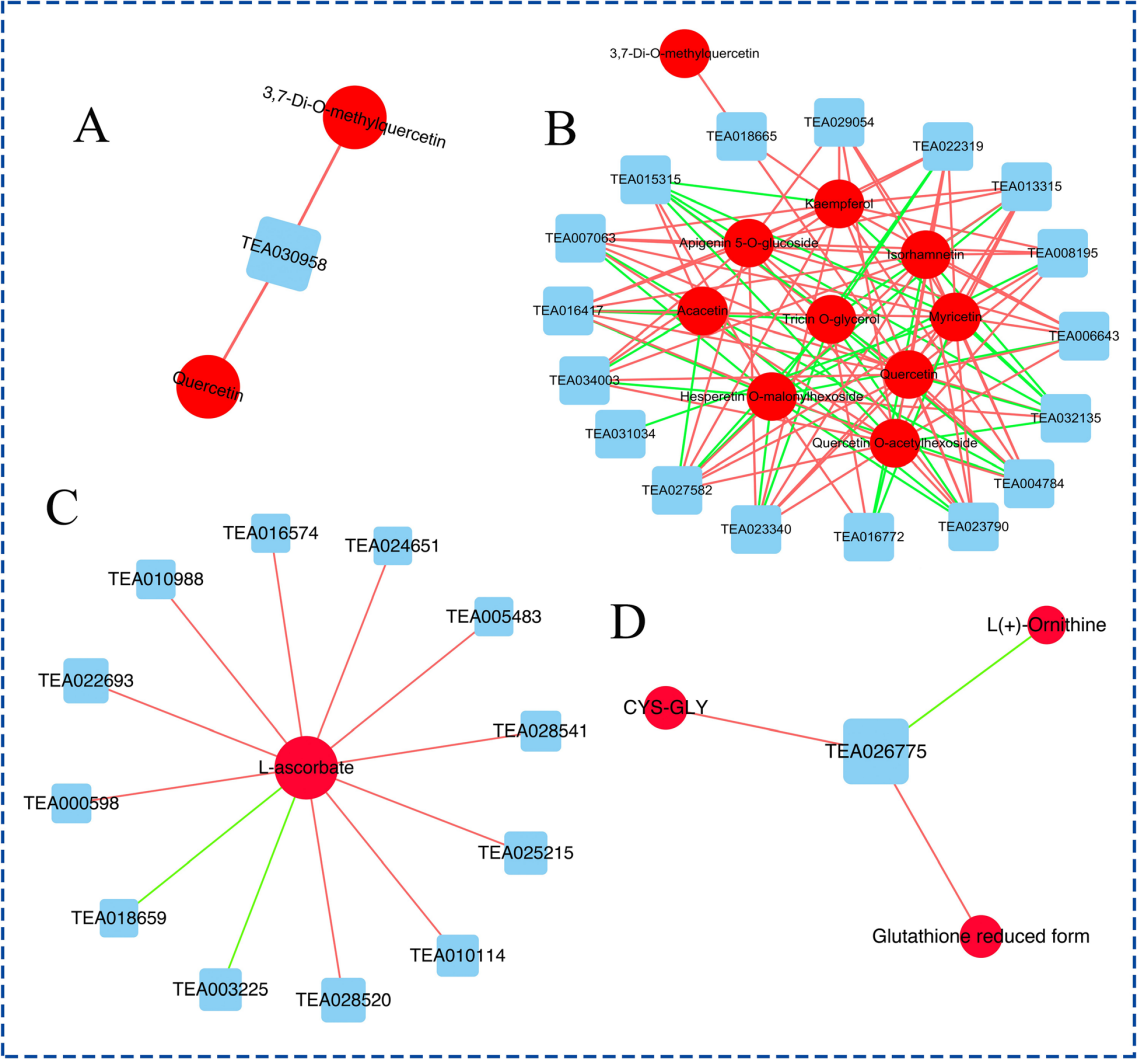
The co-expression analysis of DEGs and DEMs based on Pearson correlation. **a**. Interaction network of DEGs and DEMs involved in flavonoids biosynthesis in 4FADvs4WD. **b**. Interaction network of DEGs and DEMs associated with flavonoids biosynthesis in 8FADvs8WD. **c**. Interaction network of DEGs and DEMs involved in ascorbate metabolism in 8FADvs8WD. **d**. Interaction network of DEGs and DEMs associated with glutathione metabolism in 8FADvs8WD. Edges colored in ‘red’ and ‘green’ represent positive and negative correlations, respectively, as determined by a Pearson correlation coefficient > 0.8and or a Pearson correlation coefficient < − 0.8, respectively. TEA030958, caffeoyl-CoA O-methyltransferase; TEA018665, Chalcone synthase 3; TEA023340, Chalcone synthase 1; TEA016772, cinnamate-4-hydroxylase; TEA023790, flavanone 3-hydroxylase; TEA034003, chalcone isomerase; TEA029054, vinorine synthase; TEA027582, leucoanthocyanidin reductase; TEA016574, aldehyde dehydrogenase family 3; TEA032135, shikimate O-hydroxycinnamoyltransferase; TEA013315, flavonoid 3′,5′-hydroxylase; TEA030958, caffeoyl-CoA O-methyltransferase; TEA022397, cinnamyl alcohol dehydrogenase 3; TEA018652, L-ascorbate oxidase; TEA018659, L-ascorbate oxidase; TEA005483, GDP-mannose-3′,5′-epimerase; TEA011544, perakine reductase; TEA011287, glutathione S-transferase; TEA015341, glutathione S-transferase; TEA000526, glutathione S-transferase; TEA026775, glutathione S-transferase

In order to examine the relationship of DEGs and DEMs in FA-treated tea plants during drought stress, the co-expression network analysis of DEGs and DEMs was conducted (Pearson correlation coefficient > 0.8 or < − 0.8, *P*-value < 0.05; Additional file 9). The DEGs and DEMs in 4FADvs4WD, such as caffeoyl-CoA O-methyltransferase, Quercetin and 3,7-Di-O-methylquercetin, were found to be related to flavonoids biosynthesis (Fig. 4a). While, except for flavonoids biosynthesis (e.g. Chalcone synthase 3, Chalcone synthase 1, flavanone 3-hydroxylase, flavonoid 3′,5′-hydroxylase, Quercetin, Kaempferol, Myricetin, Phloretin and Myricetin) (Fig. 4b), the co-expression network of DEGs and DEMs in 8FADvs8WD were mainly enriched in ascorbate metabolism (e.g. L-ascorbate oxidase, aldehyde dehydrogenase family 3, GDP-mannose-3′,5′-epimerase perakine reductas, L-ascorbate) (Fig. 4c) and glutathione metabolism (e.g. glucose-6-phosphate 1-dehydrogenase 1, glutathione S-transferase, CYS-GLY and Glutathione reduced form) (Fig. 4d). The results showed that the FA could modulate the co-expression of DEGs and DEMs related to ascorbate metabolism, glutathione metabolism and flavonoids biosynthesis during drought stress.

### The integrated analysis of genes and metabolites related to ascorbate metabolism in FA-treated tea plants under drought stress

**Fig. 5:**
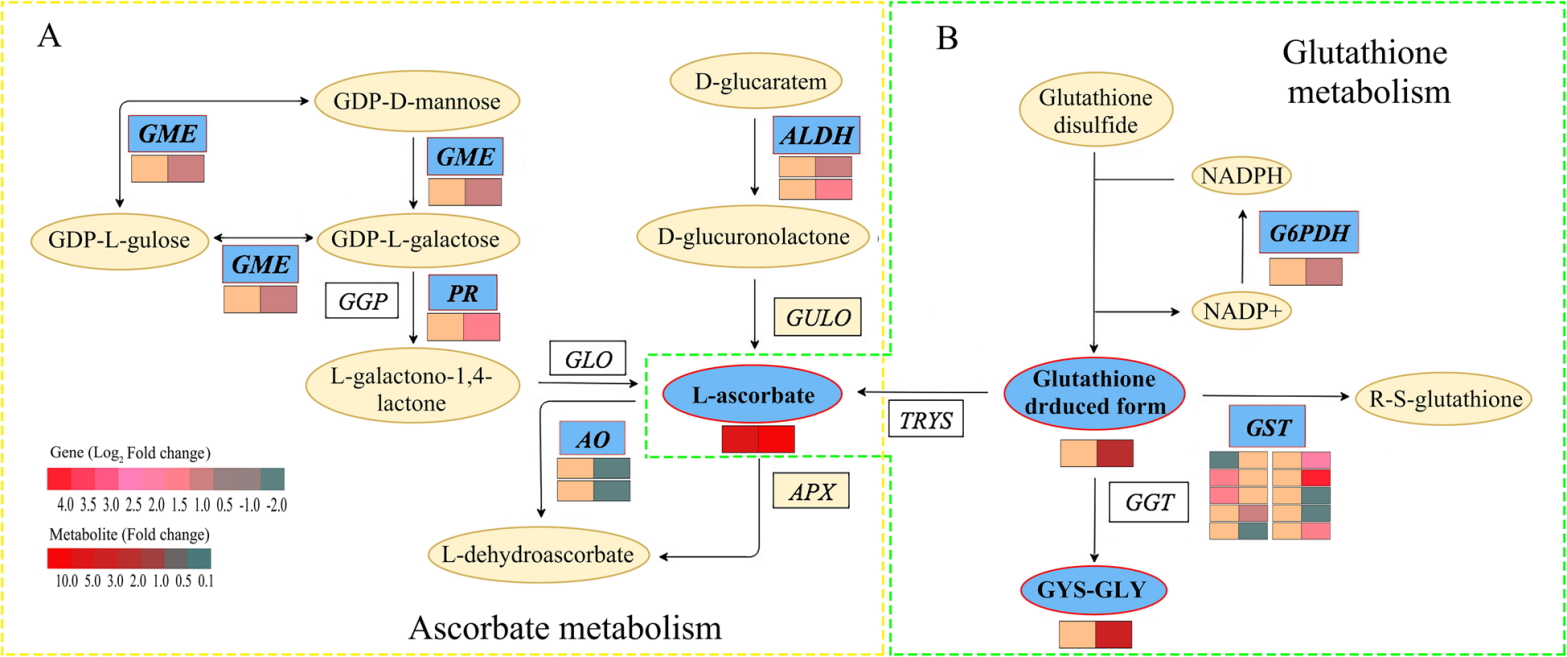
The DEGs and DEMs involved in ascorbate and glutathione metabolism in response to drought stress. **a**. The DEGs and DEMs involved in ascorbate metabolism. **b**. The DEGs and DEMs involved in glutathione metabolism. The yellow dotted box on the left represented the ascorbate metabolism process, and the green dotted box on the right represented the glutathione metabolism. The blue pattern represented the metabolites or genes that changed under drought stress. The rectangle was divided into two equal parts (the left of rectangle represented DEGs or DEMs in 4FADvs4WD; the right of rectangle represented DEGs or DEMs in 8FADvs8WD). The color in the rectangle represents the genes or metabolites were regulated under drought stress (red indicated up-regulation; yellow indicates; green indicated down-regulation). *GME*: GDP-mannose-3′,5′-epimerase; *AO*: L-ascorbate oxidase; *ALDH*: aldehyde dehydrogenase; *GST*: glutathione S-transferase; *PR*: perakine reductase; *G6PDH*: glucose-6-phosphate 1-dehydrogenase

To check into the impacts of FA on genes and metabolites related to ascorbate biosynthesis in tea plants under drought stress, the interaction of DEGs and DEMs related to ascorbate metabolism was analyzed (Fig. 5a, Additional file 10). The DEGs were not found to be related to ascorbate metabolism in 4FADvs4WD, while 8 DEGs were found in 8FADvs8WD. Of which, the DEGs, such as *GME* (GDP-mannose-3′,5′-epimerase, TEA005483), *ALDH* (aldehyde dehydrogenase family 3, TEA032018) and *PR* (perakine reductase, TEA011544) were up-regulated, and *AO* (L-ascorbate oxidase, TEA018652 and TEA018659) were down-regulated. Moreover, L-ascorbate, the downstream metabolite related to ascorbate metabolism, was found to be significantly accumulated both in 4FADvs4WD and 8FADvs8WD. The results showed that the DEGs and DEMs related to ascorbate metabolism in FA-treated tea plants were taken conjointly to respond to mild drought stress and serious drought stress.

### The integrated analysis of genes and metabolites related to glutathione metabolism in FA-treated tea plants under drought stress

To dig out the impacts of FA on expressions of genes and metabolites related to glutathione metabolism in tea plants under drought stress, the interaction of DEGs and DEMs related to glutathione metabolism was analyzed (Fig. 5b, Additional file 11). The 3 and 12 DEGs were found to be related to glutathione metabolism in 4FADvs4WD and 8FADvs8WD, respectively. Of them, 2 *GST* genes (glutathione S-transferase, TEA011287 and TEA003225) were up-regulated in 4FADvs4WD, and *GST* (TEA015341) was down-regulated. Furthermore, in 8FADvs8WD, 6 *GST* genes (TEA000526, TEA000598, TEA010114, TEA010988, TEA022693, TEA026775 and TEA028520) and *G6PDH* (glucose-6-phosphate 1-dehydrogenase 1, TEA024651) were up-regulated. Moreover, the glutathione reduced form and CYS-GLY, the downstream metabolite related to glutathione metabolism, were found to be accumulated only in 8FADvs8WD. The results showed that the DEGs and DEMs related to glutathione metabolism in FA-treated tea plants were taken conjointly to respond to mild drought stress and serious drought stress.

### The integrated analysis of genes and metabolites related to flavonoids biosynthesis in FA-treated tea plants under drought stress

**Fig. 6:**
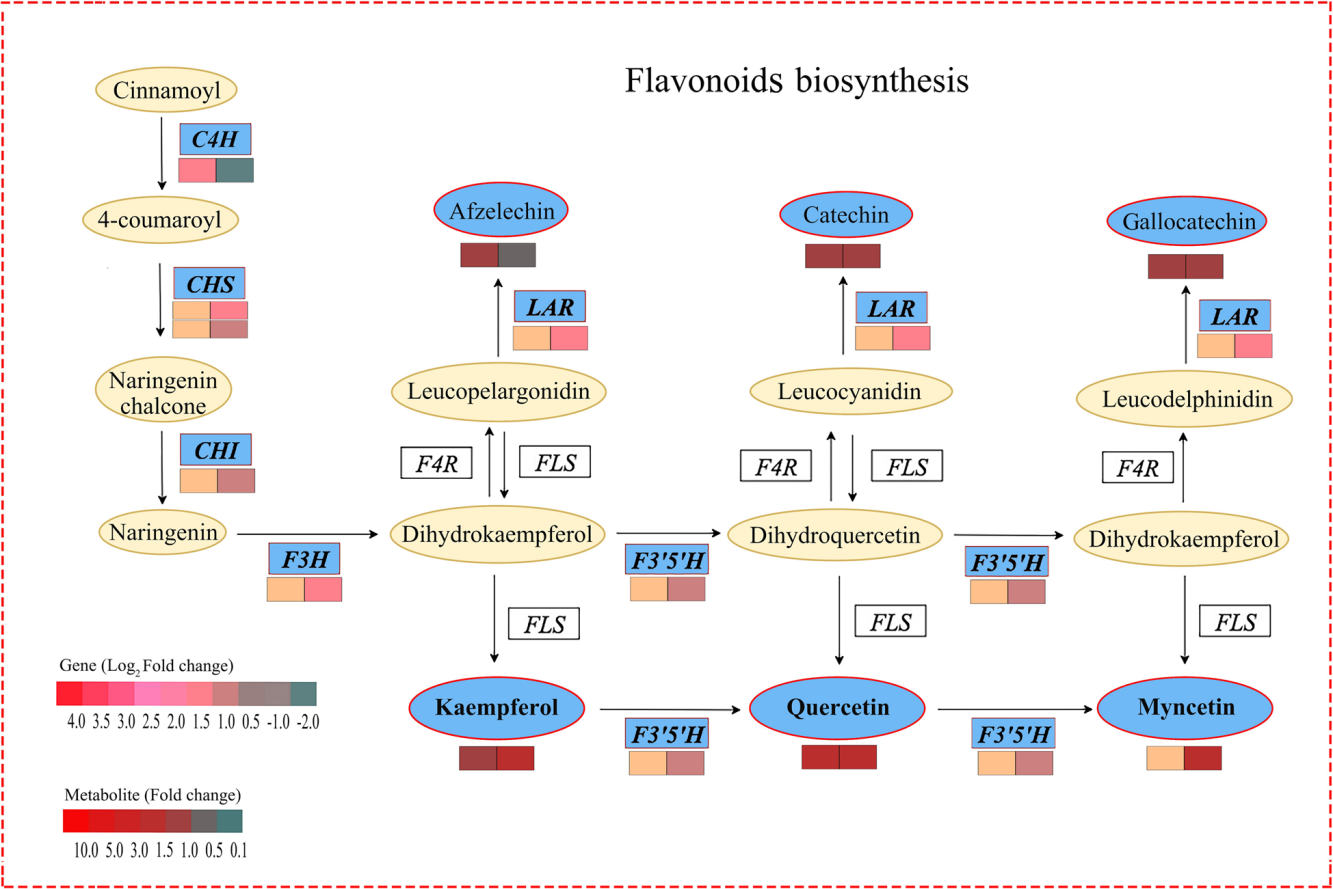
The DEGs and DEMs involved in flavonoids biosynthesis in response to drought stress. The blue pattern represented the metabolites or genes that changed under drought stress. The rectangle was divided into two equal parts (the left of rectangle represented DEGs or DEMs in 4FADvs4WD; the right of rectangle represented DEGs or DEMs in 8FADvs8WD). The color in the rectangle represents the genes or metabolites were regulated under drought stress (red indicated up-regulation; green indicated down-regulation). *C4H*: cinnamate-4-hydroxylase; *CHS*; chalcone synthase; *CHI*: chalcone isomerase; *F3H*: flavanone 3-hydroxylase; *F3’5’H*: flavonoid 3′,5′-hydroxylase; *LAR*: leucoanthocyanidin reductase

To probe into the impacts of FA on genes and metabolites related to secondary metabolism in tea plants under drought stress, the interaction of DEGs and DEMs related to flavonoids biosynthesis was analyzed (Fig. 6, Additional file 12). The 2 DEGs related to flavonoids biosynthesis, *C4H* (cinnamate-4-hydroxylase, TEA016772) and *CCM* (caffeoyl-CoA O-methyltransferase, TEA030958), were found to be up-regulated in 4FADvs4WD. Of which, the, were up-regulated. Furthermore, 20 DEGs were found in 8FADvs8WD, such as *F3’5’H* (flavonoid 3′,5′-hydroxylase, TEA013315), *LAR* (leucoanthocyanidin reductase, TEA027582), *F3H* (flavanone 3-hydroxylase, TEA023790), *CHI* (chalcone isomerase, TEA034003), *CHS1*(chalcone synthase 1, TEA023340) and *CHS*3 (chalcone synthase 3, TEA018665) were up-regulated. Moreover, the kaempferol and quercetin, the downstream metabolite related to flavonoids biosynthesis, were found to be accumulated both in 4FADvs4WD and 8FADvs8WD. And the myricetin was accumulated only in 8FADvs8WD. The results showed that the DEGs and DEMs related to flavonoids biosynthesis in FA-treated tea plants were taken conjointly to respond to mild drought stress and serious drought stress.

### The qRT-PCR validation of DEGs in FA-treated tea plants under drought stress

To test the accuracy of our RNA-Seq data, quantitative RT-PCR (qRT-PCR) was performed on 10 DEGs selected from the ascorbate metabolism, glutathione metabolism and flavonoids biosynthesis, such as *AO*, *F3H, F3’5’H* and *LAR*. The RNA-seq (FPKM) and qRT-PCR analyses were similar (Additional file 13), showing the same general expression trends (more than 90% correlation). These results verified the reproducibility and credibility of RNA-seq data.

## Discussion

In the study, to explore the roles of FA in tea plants under drought condition, we studied the effects of 0.1 g/L FA on ascorbate metabolism, glutathione metabolism and flavonoids biosynthesis by the integrated analysis of transcriptome and metabolome. The results showed that FA could ameliorate drought stress-induced damage in tea plants.

### FA enhanced the ascorbate metabolism in tea plants under drought stress

The drought stress often causes rapid and excessive accumulation of *ROS* in plant cells, which resulted in degradation of chlorophyll, lipid peroxidation, enzyme inactivation and DNA damage, and eventually led to metabolic disorder and apoptosis [20, 21]. To protect the plants from oxidative stress and maintain normal cellular functions, plants in drought condition would scavenge excess *ROS* through *ROS* scavenging enzymes (*CAT*, *POD* and *SOD*) and non-enzymatic antioxidants (including ascorbate and glutathione) [22, 23]. Interestingly, in the present study, the *SOD*, *CAT* and *POD* did not show significant statistical difference (*P* > 0.05) at the level of transcription. The results hinted that FA might improve the *ROS* scavenging of tea plants mainly by enhancing the biosynthesis of non-enzymatic antioxidants.

Ascorbic acid functions as an antioxidant and enzymatic cofactor and plays effective roles in maintenance of *ROS* homeostasis [24]. However, up to now, the ascorbate in FA-treated plants under drought stress has not been reported. In this study, the contents of ascorbate were accumulated both in 4FADvs4WD and 8FADvs8WD, and the statistically differences under serious drought stress were severe (fold changes > 30). In addition, the *GME* and *AO* were up-regulated and down-regulated in 8FADvs8WD, respectively. The *GME*, generally considered to be a central enzyme of ascorbate biosynthesis pathway, catalyzed the conversion of GDP-D-mannose to GDP-L-galactose in the D-mannose/L-galactose pathway [25]. The *AO* could catalyze the oxidation of ascorbate to the unstable radical monodehydroascorbate which rapidly disproportionated to yield dehydroascorbate [26]. Previous studies showed that the *GME* could effectively enhance tolerance of transgenic *Arabidopsis* to drought by increasing ascorbate accumulation [25]. The *AO* was involved in the negative regulation of the stress response, and *AO* caused oxidation of ascorbate, thereby preventing the detoxification of *ROS* [27]. Therefore, we deduced that FA might enhance the *ROS* homeostasis of tea plants during drought stress by improving the biosynthesis of ascorbate, while further enhance the *ROS* homeostasis under serious drought stress by improving the expression of *GME* and restraining the expression of *AO*.

### FA enhanced the glutathione metabolism in tea plants under drought stress

Glutathione is one of the major endogenous antioxidants in plants, which functions as a substrate in antioxidative defense mechanisms by conjugating to toxic electrophilic compounds, scavenging free radicals, and reducing peroxides [28, 29]. Previous studies showed the *G6PDH* was involved in glutathione content maintenance and *ROS* scavenging under drought stress by utilizing NADPH [30]. The expression of *G6PDH* in tomato could control the pace of *ROS* increment and improve the drought tolerance [31]. In this study, the *G6PDH* and glutathione reduced form were accumulated only in 8FADvs8WD, indicating that the FA might improve the *ROS* scavenging in tea plants under serious drought stress by improving the expression of *G6PDH* and the biosynthesis of glutathione reduced form. In addition, previous studies showed that *GST* was enzyme that utilize glutathione to play an important role in plant defense metabolism by mechanisms that aid in the reduction of secondary noxious products resulting from exposure to stress-induced *ROS* [28, 32]. And the over-expressing *GST* improved drought tolerance in transgenic *Arabidopsis* [33]. In the study, we also found that *GST* genes were up-regulated both in 4FADvs4WD and 8FADvs8WD. Therefore, we speculated that FA might reduce the secondary injury products of *ROS* under drought stress through the expression of *GST*.

### FA improved the flavonoids biosynthesis in tea plants under drought stress

Flavonoids are a group of polyphenol compounds with known antioxidant activities, which are composed of flavones, flavonols, flavanones, anthocyanins and isoflavones [34]. It has been suggested that the accumulation of flavonoids could be a key step in development of plant tolerance to different environmental stresses [35]. The *C4H*, a member of the cytochrome P450 monooxygenase superfamily, controlled the synthesis of p-coumaric acid from trans-cinnamic acid [36]. The *CHS* catalyzed the condensation of three molecules of malonyl-CoA and one molecule of 4-coumaroyl-CoA to naringenin chalcone, which was the substrate for *CHI* and was converted to naringenin [37]. Earlier researches revealed that the *C4H* involved in the drought defense of cucumber [38]. And the *CHS* from *Abelmoschus esculentus* regulated flavonoid accumulation and abiotic stress tolerance in transgenic *Arabidopsis* [39]. In this study, we found that the *C4H* and 2 *CHS* genes (*CHS*1 and *CHS*3) were up-regulated in 4FADvs4WD and 8FADvs8WD, respectively. Therefore, we speculated that FA could modulate the drought tolerance of tea plants during drought stress by improving the expression of *C4H* and *CHS* in mild drought and serious drought, respectively.

The main physiological functions of quercetin, kaempferol and myricetin in plants were scavenging reactive oxygen species and acting to detoxification of free radicals, which increasing tolerance to adapt to environmental change [40–42]. Previous study showed that the accumulation of quercetin and kaempferol under water deficit could improve the drought tolerance of white clover [43]. However, the information of flavonoids in FA-treated plants under drought stress was rare. In this study, the flavonoids, including kaempferol, quercetin and myricetin, were accumulated during drought stress, and the corresponding genes, including *F3’H* and *F3’5’H*, were up-regulated in 8FADvs8WD. Previous studies showed that the *F3’H* and *F3’5’H* played a key role in affecting the composition of dihydroxylated and trihydroxylated flavonoids [44]. And the lack of expression of the *F3’5’H* in the grapes restricted the presence of quercetin, kaempferol, myricetin and syringetin derivatives [45]. Thus, we have reason to deduce that the FA might enhance the *ROS* scavenging by improving the biosynthesis of kaempferol, quercetin and myricetin during drought stress, while further enhance *ROS* scavenging by the expression of *F3’H* and *F3’5’H* under serious drought stress.

The *LAR* catalyzes the conversion of leucocyanidin, leucodelphinidin or leucopelargonidin to the corresponding flavan-3-ol units (catechin and gallocatechin) [46]. And the overexpression of *LAR* in plants often results in the increases of catechin and epicatechin [47]. According to previous report in berry, the soil drought could induce the expression of *LAR* genes, resulted in the accumulation of catechins [48]. The results from the current paper indicated that the *LAR* was up-regulated in 8FADvs8WD, and the catechin and gallocatechin were also slightly increased in FA-treated tea plants. Since previous studies have shown that amongst flavonoids, flavan-3-ols (catechin, epicatechin and gallocatechin) have direct free radical scavenging activity to maintain the normal physiological function of cells in vivo [49]. So, these data meant that FA might contribute to the drought acclimation of tea plants under serious drought condition by improving the expression of *LAR* and the biosynthesis of catechin and gallocatechin.

## Conclusion

In the present study, we investigated the positive roles of FA in tea plants under drought stress. The novel regulatory and functional candidates in ascorbate metabolism, glutathione metabolism and flavonoids biosynthesis in FA-treated tea plants in providing drought tolerance were successfully identified by comparative transcriptional and metabolic analysis. Our study confirmed that the primary strategy for FA to protect tea plants against drought-stress was to enhance the detoxifying *ROS* and regulate the antioxidant systems.

## Methods

### Plant material and experimental design

The uniformly sized one-year-old seedlings of *C.sinensis* (L.) O. Kuntze cv. ‘QN3’ [50], acquired from Qingdao Agricultural University in Shandong Province of China (36°19′N, 120°23′E), were transplanted into plastic cups filled with sandy loam soil and pH 4.5. The ‘QN3’ was identified by Prof Zhaotang Ding, Yu wang and etc. And it has been deposited in the national germplasm tea repository (Hangzhou, China). The seedlings were culture-grown under a 12 h light (25 °C) / 12 h dark (18 °C) photoperiod with 300 μM•m-2•s-1 photon flux densities and 75% humidity. After 1 week, the tea plants in plastic cups subjected to drought stress. The preliminary experiments for FA concentration screening (0.01, 0.05, 0.1, 0.5 and 1 g/L FA) were found that 0.1 g/L FA was better concentration for alleviating drought damage during water deficit (See Additional file 14 for a detailed description). The FA was purchased from Bio Dibai (Shanghai, China). In the formalized testing, tea plants in plastic cups were exposed to drought stress after irrigating 15 ml of 0.1 g/L FA, and the same water was irrigated as the control. The soil water content in the normal condition was at 65–75% water holding capacity, the severe drought condition was at 15–25% water holding capacity [51]. The *LWC*, *REL* and *ROS* contents of tea new shoots (the sprout and the two leaves below) were assayed during drought stress. Based on the results of trials, the tea shoots were harvested at 4 (mild drought stress) and 8 (severe drought stress) days of drought stress for analyzing the transcriptome and metabolome. Three biological replicates for each sampling time point were collected and immediately stored at − 80 °C.

### Measurement of physiological indices of drought stress

In physiological experiments, more than five tea plants were harvested for each treatment group at 0, 2, 4, 6, 8, and 10 days. The tea shoots were used to test the physiological traits containing *LWC*, *ELC* and *ROS*. The *LWC* and *ELC* were determined as described previously [52, 53]. The *ROS* was tested using ELISA kit (Jiangsu Meimian, China).

### Transcriptome sequencing and data analysis

The samples of 4FAD, 4WD, 8FAD and 8WD were selected for total RNA extract, which using the Trizol reagent (Invitrogen, USA). Refer to previous study for transcriptome sequencing [54]. Briefly, RNA concentrations were measured by Qubit 2.0 Flurometer (Life Technologies, USA). The cDNA library was prepared for sequencing using the NEBNext Ultra RNA Library Prep Kit. The prepared library was sequenced using novaseq 6000 platform. Reads containing adapters, sequences with more than 10% unknown nucleotides (N), and a quality rating less than 50% (Q-value ≤20) were first removed from the data set. Index of the reference genome was built using Bowtie v2.2.3 and paired-end clean reads were aligned to the tea plant reference genome (http://tpia.teaplant.org/) using TopHat (v2.0.12). HTSeq v0.6.1 was used to count the reads numbers mapped to each gene. And then FPKM of each gene was calculated based on the length of the gene and reads count mapped to this gene. Differential expression between two conditions/groups was analysed with the DESeq R package (1.10.1). Genes with |log2FC| > 1 and *p*-value < 0.05 were considered as DEGs. To infer the putative functions of DEGs, we conducted GO functions and KEGG pathway analysis by the OmicShare tools (http://www.omicshare.com/tools).

### Metabolite profiling analysis

The extract analysis, metabolite identification and quantification were performed by MetWare (Wuhan, China) following their standard procedures and previous study [55]. Metabolite data analysis was conducted with the Analyst 1.6.3 software. Metabolites with fold change ≥1.5 were considered as DEMs.

### Co-expression network analysis of metabolome and transcriptome

The EXCEL program was used to calculate the Pearson correlation coefficients according to the fold changes of each DEGs and each DEMs. Correlations corresponding to a coefficient with R^2^ > 0.8 were selected. The Cytoscape (version 2.8.2) was used to visualize the relationship between metabolome and transcriptome.

### The qRT-PCR validation for DEGs

The qRT-PCR analyses were conducted with mRNA from the tea shoots. The method followed the procedures described in a previous report [56]. The triplicates of each reaction were performed, and *GAPDH* sequence was used as endogenous control. CT values obtained through qRT-PCR were analyzed using 2^-ΔCT^ method to calculate relative fold change values [57]. The primers for the qRT-PCR are described in Additional file 15.

## Supplementary information

**Additional file 1.** The water holding capacity of soil.

**Additional file 2.** The information of transcriptomes.

**Additional file 3.** The annotation of total reads.

**Additional file 4.** The information of DEGs in 4FADvs4WD and 8FADvs8WD.

**Additional file 5.** The information of GO and KEGG analysis of DEGs.

**Additional file 6.** The information of metabolomes.

**Additional file 7.** The information of DEMs in 4FADvs4WD and 8FADvs8WD.

**Additional file 8.** The summary of DEMs in 4FADvs4WD and 8FADvs8WD.

**Additional file 9.** The information of co-expression network of DEGs and DEMs.

**Additional file 10.** The information of DEGs and DEMs involved in ascorbate metabolism.

**Additional file 11.** The information of DEGs and DEMs involved in glutathione metabolism.

**Additional file 12.** The information of DEGs and DEMs involved in flavonoids biosynthesis.

**Additional file 13.** The qRT-PCR validation of 10 genes.

**Additional file 14.** The detailed description for concentration screening of fulvic acid under drought stress.

**Additional file 15.** The information of primers used for qRT-PCR.

## Data Availability

The datasets supporting the results of this article are available at the Sequence ReadArchive (SRA) database of National Center for Biotechnology Information (NCBI; https://www.ncbi.nlm.nih.gov/) under project accession number PRJNA596070.

## Abbreviations

FA: Fulvic acid
*SOD*: Superoxide dismutase
*POD*: Peroxidase
*CAT*: Catalase
*LWC*: Relative water content
*CC*: Chlorophyll content
*ELC*: Relative electrolyte conductivity
*ROS*: Reactive oxygen species
FAD: FA-treated tea plants were exposed to drought stress
4FAD: FA-treated tea plants at 4 days of drought stress
8FAD: FA-treated tea plants at 8 days of drought stress
4WD: The controlled groups at 4 days of drought stress
8WD: The controlled groups at 8 days of drought stress
LC-MS/MS: liquid chromatography tandem mass spectrometry
RNA-seq: RNA sequencing
GO: Gene Ontology
KEGG: Kyoto Encyclopedia of Genes and Genomes
DEGs: Differentially expressed genes
DEMs: Differentially expressed metabolites
qRT-PCR: Quantitative RT-PCR
*GME*: GDP-mannose-3′,5′-epimerase
*AO*: L-ascorbate oxidase
*ALDH*: Aldehyde dehydrogenase
*PR*: Perakine reductase
*G6PDH*: Glucose-6-phosphate 1-dehydrogenase
*GST*: Glutathione S-transferase
*C4H*: Cinnamate-4-hydroxylase
*CHS*: Chalcone synthase
*CHI*: Chalcone isomerase
*F3H*: Flavanone 3-hydroxylase
*F3’5’H*: Flavonoid 3′,5′-hydroxylase
*LAR*: Leucoanthocyanidin reductase
