# Supplementary material for: Fulvic acid ameliorates drought stress-induced damage in tea plants by regulating the ascorbate metabolism and flavonoids biosynthesis
Source: BMC Genomics. 2020 Jun 18;21:411. doi: 10.1186/s12864-020-06815-4 (PMC7301537; doi:10.1186/s12864-020-06815-4)
Supplement: Supplementary file 1 — Additional file 1. The water holding capacity of soil. [file 12864_2020_6815_MOESM1_ESM.docx]

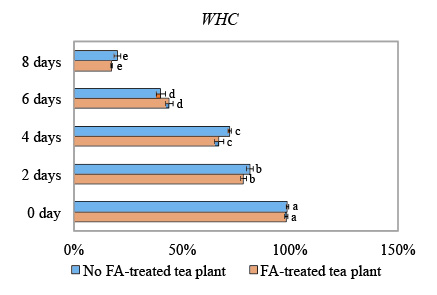


**Figure S1.** The water holding capacity of soil (*WHC*) traits of FA-treated tea plants under drought stress.


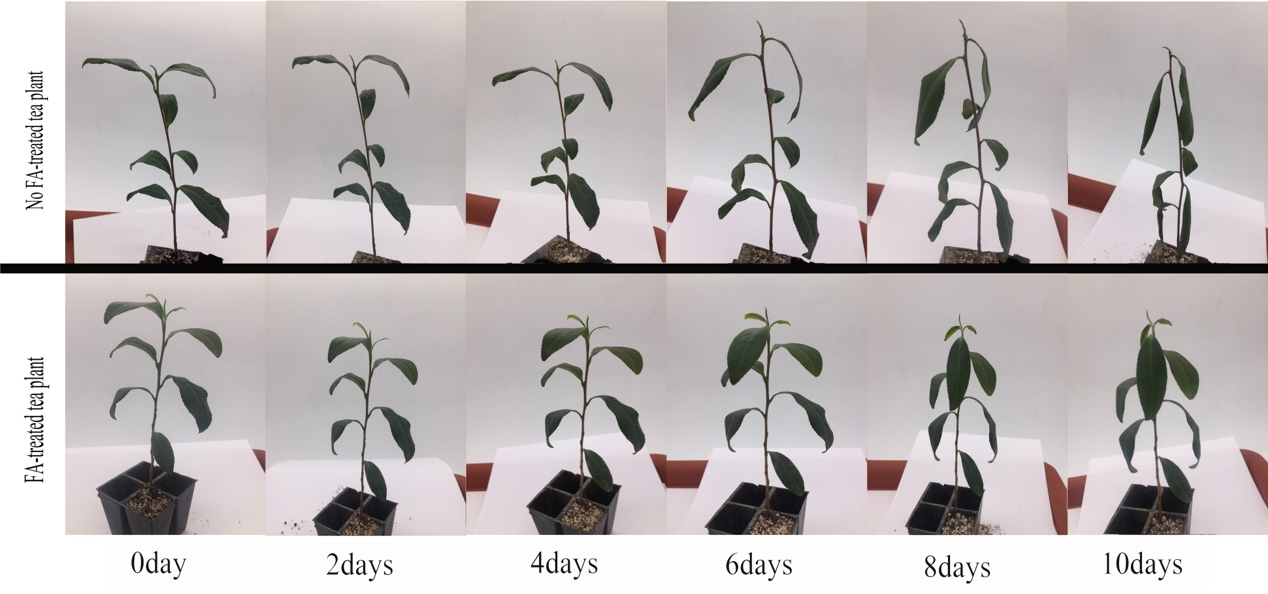


**Figure S2.** The original images.
