# Supplementary material for: Fulvic acid ameliorates drought stress-induced damage in tea plants by regulating the ascorbate metabolism and flavonoids biosynthesis
Source: BMC Genomics. 2020 Jun 18;21:411. doi: 10.1186/s12864-020-06815-4 (PMC7301537; doi:10.1186/s12864-020-06815-4)
Supplement: Supplementary file 13 — Additional file 13. The qRT-PCR validation of 10 genes. [file 12864_2020_6815_MOESM13_ESM.docx]

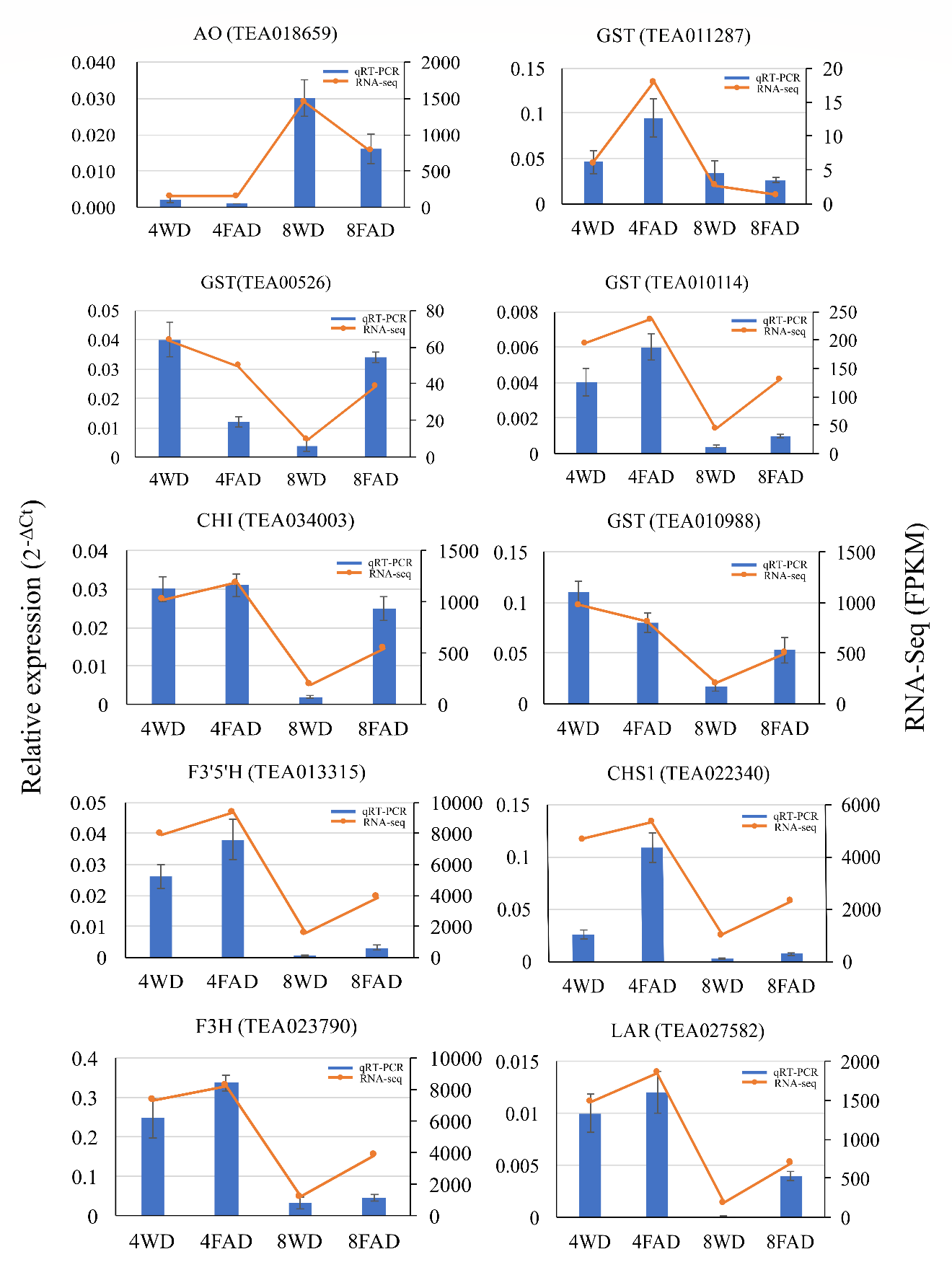


**Fig.1 The qRT-PCR validation of 10 genes.** *AO*, L-ascorbate oxidase; *GST*, glutathione S-transferase. *CHS*, chalcone synthase; *CHI*, chalcone isomerase; *F3H*, flavanone 3-hydroxylase; *F3’5’H*, flavonoid 3',5'-hydroxylase; *LAR*, leucoanthocyanidin reductase.
