# Supplementary material for: Fulvic acid ameliorates drought stress-induced damage in tea plants by regulating the ascorbate metabolism and flavonoids biosynthesis
Source: BMC Genomics. 2020 Jun 18;21:411. doi: 10.1186/s12864-020-06815-4 (PMC7301537; doi:10.1186/s12864-020-06815-4)
Supplement: Supplementary file 14 — Additional file 14. The detailed description for concentration screening of fulvic acid under drought stress. [file 12864_2020_6815_MOESM14_ESM.docx]

### Materials and Methods

# Plant material and experimental design

The uniformly sized 1-year-old seedlings of tea plants, acquired from Tea Research Institute, Qingdao Agricultural University in Shandong Province of China, were transplanted into plastic cups (6cm in diameter, 7cm in depth) filled with sandy loam soil and pH 4.5. The growth conditions were set as follows: temperature, 25/18°C (12h day/12h night); lighting, 300 μM•m^-2^•s^-1^ photon flux densities; and humidity, 75％ relative humidity. The fulvic acid (FA) was purchased from Bio Dibai (Shanghai, China).

After the tea plants were irrigated with different concentrations of 40ml FA solution, including 0.01, 0.05, 0.1, 0.5g/L FA, the seedlings exposed to drought stress. Seedling were irrigated with distilled water as the control. All treatments were described as (1) 0 FA (control), (2) 0.01FA, (3) 0.05FA, (4) 0.1FA, (5) 0.5FA, (6)1.0FA. After 6 days of drought stress, the indicators of tea shoots were measured, including relative leaf water contents (LWC), leaf maximum photochemical quantum yield of PS II (Fv/Fm) and chlorophyll content (CC).

# Physiological determinations

For physiological experiments, more than ten tea plants were harvested and pooled for each treatment group after 6 days of drought stress, and the collection was repeated three times as biological replicates. The tea shoots were used to test the physiological traits containing the *LWC*, *Fv/Fm* and *CC*. The *LWC* and *CC* of tea shoots was determined as described previously, respectively ^[1]^. The tea leaves were subjected to a FluorCam 700mf (Photon Systems instruments, Brno, Czech Republic) on the Fo, Fm, and Kautsky effect setting to obtain maximum photochemical quantum yield of PS II (Fv/Fm = (Fm - Fo)/Fm).

## Results

In order to obtain more representative data, the proper concentrations of FA were determined under drought stress in tea plants by recording the phenotypic and physiological indicators (Fig.1). We found that 0FA-treated tea plants showed obvious wilting at 6 days of drought stress. The laminas wilting coincided with decrease in *LWC*, Fv/Fm and *CC*. Furthermore, FA-treated (especially 0.1FA) tea plants had significantly higher *LWC,* Fv/Fm and *CC* compared with the 0FA-treated tea plants. These results showed that FA might improve the ability of tea plants to resist drought stress, especially 0.1FA.


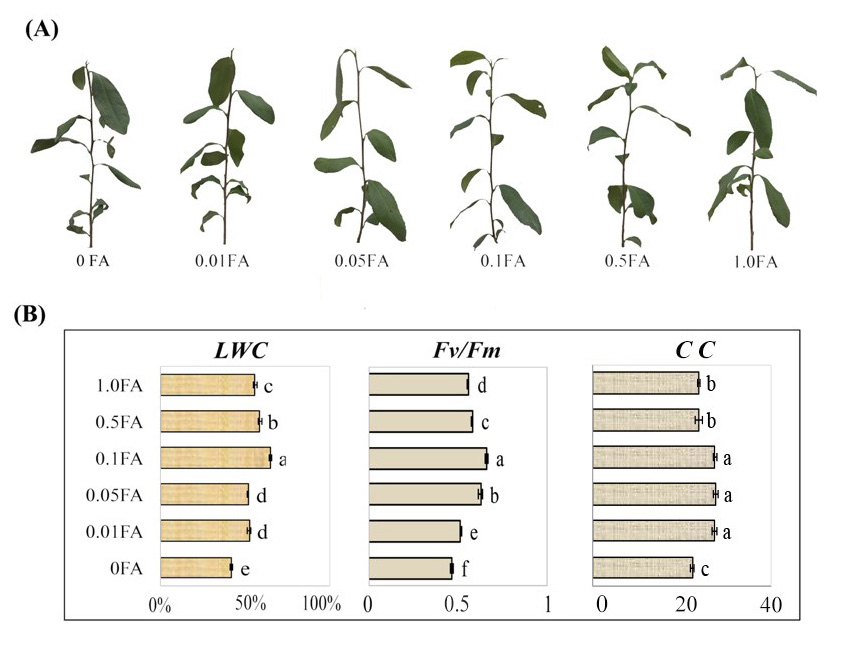


**Fig. S1** **Phenotypic and physiological** **traits of fulvic acid (FA)-treated tea plants to drought stress.** A. Phenotypes of tea plants treated with different concentrations of FA. B. The values of leaf water contents (LWC), leaf maximum photochemical quantum yield of PS II (Fv/Fm) and chlorophyll content (CC).

**References**

[1] Xie, X., Kang, H., Liu, W. & Wang, G. L. Comprehensive profiling of the rice ubiquitome reveals the significance of lysine ubiquitination in young leaves. Journal of Proteome Research 14, 2017–2025, https://doi.org/10.1021/pr5009724 (2015).
